# Supplementary material for: Causal network inference from gene transcriptional time-series response to glucocorticoids
Source: PLoS Comput Biol. 2021 Jan 29;17(1):e1008223. doi: 10.1371/journal.pcbi.1008223 (PMC7875426; doi:10.1371/journal.pcbi.1008223)
Supplement: S1 Fig — Panels show each inference method applied to a cause gene g’ (blue, solid) and an effect gene g (blue, dotted). A) Mutual information is computed between the cause and effect. B) The effect’s expression is fit as an autoregression from the cause’s past expression. C) The effect’s expression is fit as a differential equation from the cause’s current expression. D) The effect’s expression is fit as a decision tree function of the cause’s past expression. E) The space of dynamic causal networks is searched, with linear relationships between cause and effect. F) The space of dynamic causal networks is searched, with nonlinear relationships between cause and effect. (PDF) [file pcbi.1008223.s001.pdf]

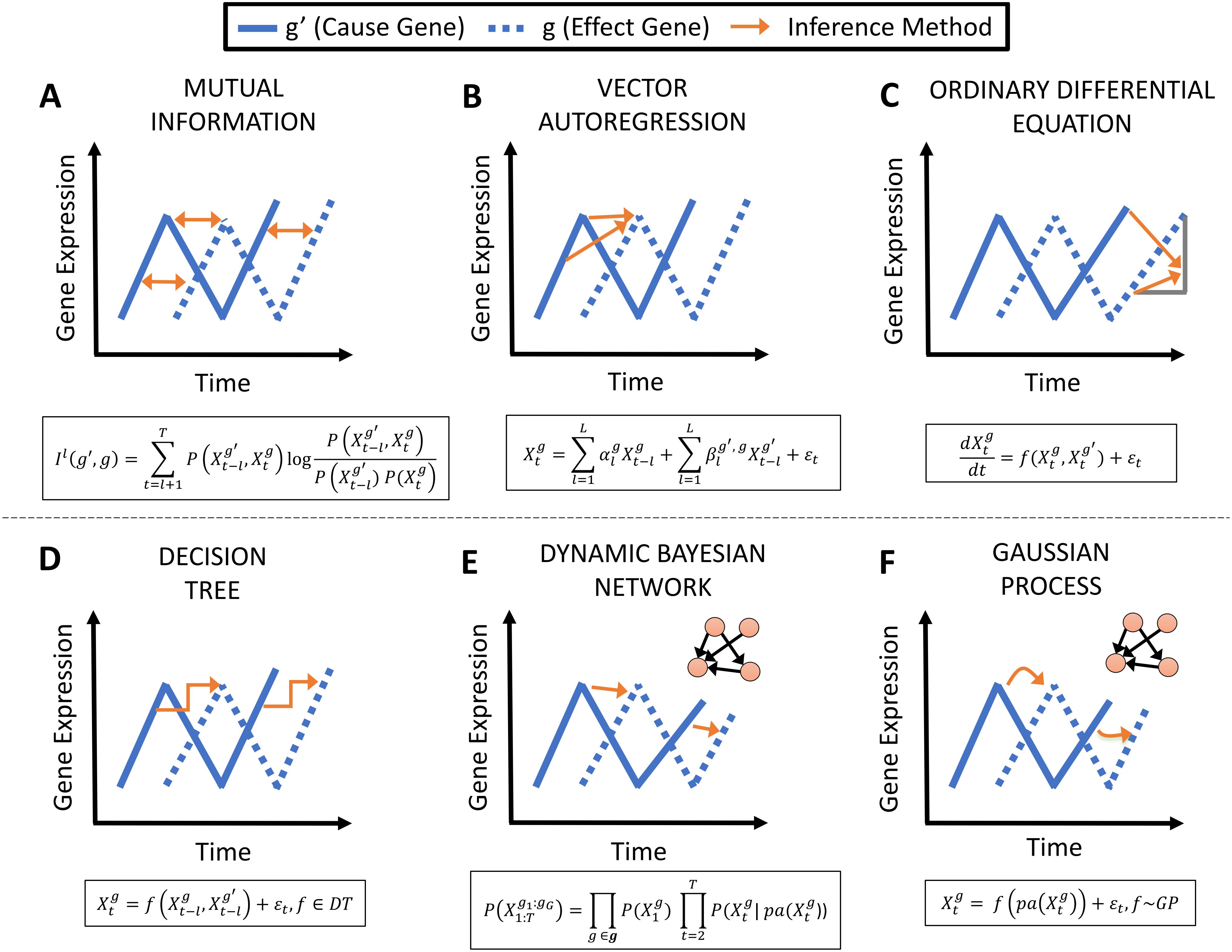
g’ (Cause Gene)
g (Effect Gene)
Inference Method
A
MUTUAL INFORMATION
Gene Expression
Time
$I^l(g',g) = \sum_{t=l+1}^{T} P\left(X_{t-l}^{g'}, X_t^g\right) \log \frac{P\left(X_{t-l}^{g'}, X_t^g\right)}{P\left(X_{t-l}^{g'}\right) P(X_t^g)}$
B
VECTOR AUTOREGRESSION
Gene Expression
Time
$X_t^g = \sum_{l=1}^{L} \alpha_l^g X_{t-l}^g + \sum_{l=1}^{L} \beta_l^{g',g} X_{t-l}^{g'} + \varepsilon_t$
C
ORDINARY DIFFERENTIAL EQUATION
Gene Expression
Time
$\frac{dX_t^g}{dt} = f(X_t^g, X_t^{g'}) + \varepsilon_t$
D
DECISION TREE
Gene Expression
Time
$X_t^g = f\left(X_{t-l}^g, X_{t-l}^{g'}\right) + \varepsilon_t, f \in DT$
E
DYNAMIC BAYESIAN NETWORK
Gene Expression
Time
$P\left(X_{1:T}^{g_1:g_G}\right) = \prod_{g \in \boldsymbol{g}} P(X_1^g) \prod_{t=2}^{T} P(X_t^g \mid pa(X_t^g))$
F
GAUSSIAN PROCESS
Gene Expression
Time
$X_t^g = f\left(pa(X_t^g)\right) + \varepsilon_t, f \sim GP$
