# Supplementary figures and images for: Causal network inference from gene transcriptional time-series response to glucocorticoids
Source: PLoS Comput Biol. 2021 Jan 29;17(1):e1008223. doi: 10.1371/journal.pcbi.1008223 (PMC7875426; doi:10.1371/journal.pcbi.1008223)

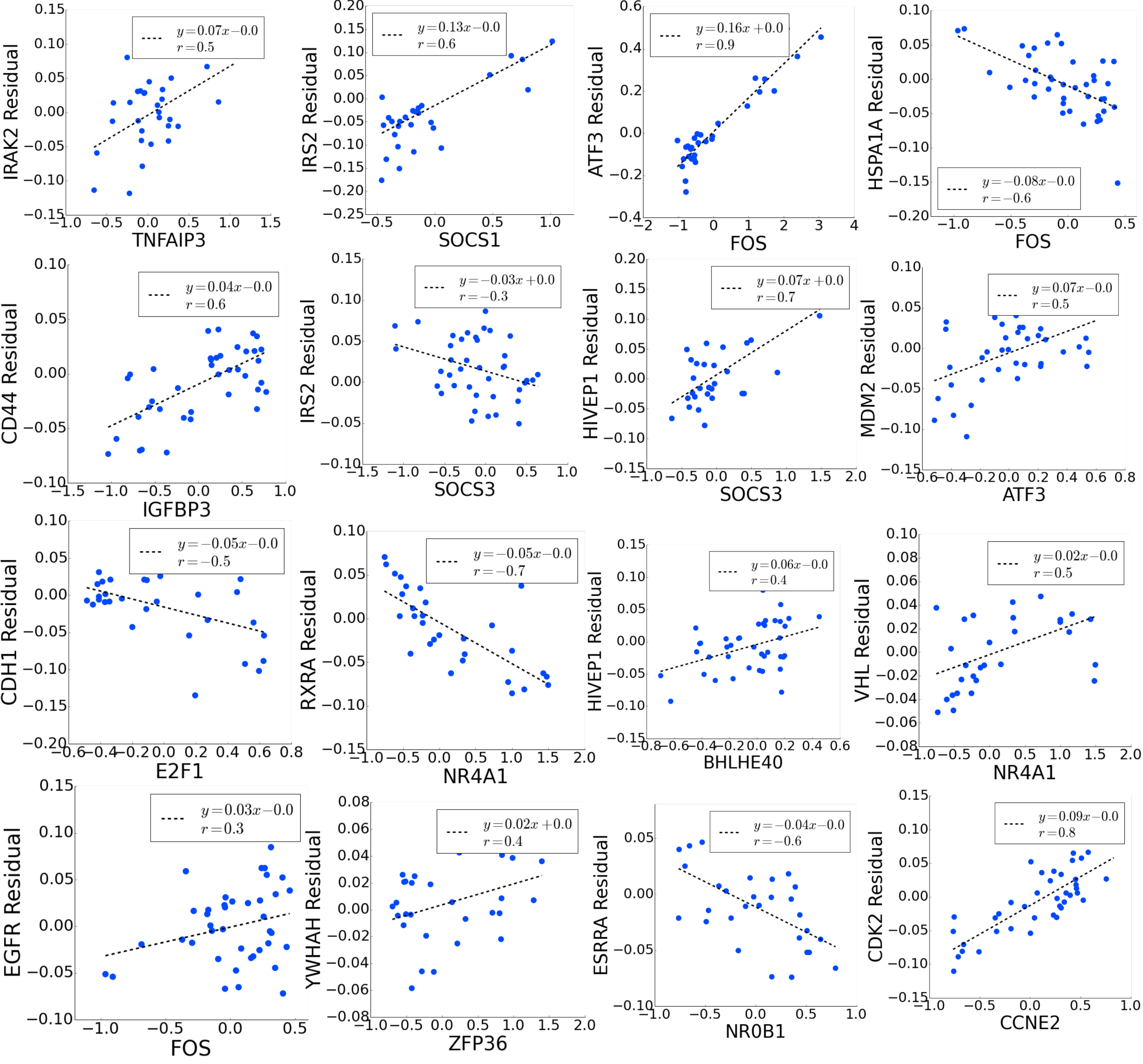

Supplement: S2 Fig — On the y-axes are the effect gene residual expression values after subtracting the effects of all other covariates. Axes are in units of ln(TPM). Related to Fig 5 and S7 Table. (PDF) [file pcbi.1008223.s002.pdf]
