# Supplementary material for: Causal network inference from gene transcriptional time-series response to glucocorticoids
Source: PLoS Comput Biol. 2021 Jan 29;17(1):e1008223. doi: 10.1371/journal.pcbi.1008223 (PMC7875426; doi:10.1371/journal.pcbi.1008223)
Supplement: S1 Table — DBN is dynamic Bayesian network, DT is decision tree, GP is Gaussian process, MI is mutual information, ODE is ordinary differential equation, VAR is vector autoregression. The references that reported ebdbnet, ScanBMA, and LASSO did not provide AUPR values for individual networks. Algorithms that were run in-house were ARACNE, BETS, CLR, CSId, Enet, Jump3, MRNET, SWING-Lasso, and SWING-RF. Where reported literature values were available, they were consistent with these values. Values for CSIc, G1DBN, GCCA, GP4GRN, TSNI, VBSSMa and VBSSMb were taken from [49]. Values for ebdnet, LASSO and ScanBMA, were taken from [40]. Values for dynGENIE3, GENIE3, OKVAR-Boost and tl-CLR were taken from [37]. Values for Inferelator and Jump3 were taken from [36]. Related to Fig 2. (DOCX) [file pcbi.1008223.s003.docx]

**S1 Table. DREAM4 100-gene Network Inference Results, AUPR.** DBN is Dynamic Bayesian Network, DT is Decision Tree, GP is Gaussian Process, MI is Mutual Information, ODE is Ordinary Differential Equation, VAR is Vector Autoregression. The references that reported ebdbnet, ScanBMA, and LASSO did not provide AUPR values for individual networks. Algorithms that were run in-house were ARACNE, BETS, CLR, CSId, Enet, Jump3, MRNET, SWING-Lasso, and SWING-RF. Where reported literature values were available, they were consistent with these values.

**­**

| **Algorithm** | **Method Type** | **AUPR (Average)** | **AUPR (STD)** | **Network 1 AUPR** | **Network 2 AUPR** | **Network 3 AUPR** | **Network 4 AUPR** | **Network 5 AUPR** |
| --- | --- | --- | --- | --- | --- | --- | --- | --- |
| ebdbnet | DBN | 0.043 |  |  |  |  |  |  |
| G1DBN | DBN | 0.11 | 0.01 | 0.11 | 0.1 | 0.13 | 0.1 | 0.11 |
| ScanBMA | DBN | 0.101 |  |  |  |  |  |  |
| VBSSMa | DBN | 0.086 | 0.02 | 0.08 | 0.05 | 0.11 | 0.1 | 0.09 |
| VBSSMb | DBN | 0.096 | 0.03 | 0.09 | 0.06 | 0.12 | 0.12 | 0.09 |
| dynGENIE3 | DT | 0.198 | 0.05 | 0.22 | 0.14 | 0.25 | 0.22 | 0.16 |
| GENIE3 | DT | 0.072 | 0.02 | 0.05 | 0.06 | 0.1 | 0.06 | 0.09 |
| Jump3 | DT | 0.182 | 0.05 | 0.26 | 0.11 | 0.19 | 0.17 | 0.18 |
| SWING-RF | DT | 0.212 | 0.05 | 0.26 | 0.14 | 0.24 | 0.22 | 0.2 |
| CSIc | GP | 0.07 | 0.04 | 0.13 | 0.03 | 0.07 | 0.07 | 0.05 |
| CSId | GP | 0.208 | 0.03 | 0.26 | 0.17 | 0.22 | 0.2 | 0.19 |
| GP4GRN | GP | 0.162 | 0.05 | 0.22 | 0.1 | 0.16 | 0.21 | 0.12 |
| ARACNE | MI | 0.046 | 0.01 | 0.03 | 0.04 | 0.06 | 0.04 | 0.06 |
| CLR | MI | 0.072 | 0.02 | 0.05 | 0.06 | 0.11 | 0.06 | 0.08 |
| MRNET | MI | 0.068 | 0.02 | 0.04 | 0.06 | 0.1 | 0.06 | 0.08 |
| tl-CLR | MI | 0.168 | 0.05 | 0.18 | 0.11 | 0.24 | 0.15 | 0.16 |
| Inferelator | ODE | 0.0688 | 0.01 | 0.063 | 0.071 | 0.075 | 0.073 | 0.062 |
| TSNI | ODE | 0.026 | 0.01 | 0.02 | 0.03 | 0.03 | 0.02 | 0.03 |
| BETS | VAR | 0.128 | 0.02 | 0.16 | 0.1 | 0.13 | 0.14 | 0.11 |
| Enet | VAR | 0.098 | 0.02 | 0.12 | 0.08 | 0.1 | 0.11 | 0.08 |
| GCCA | VAR | 0.05 | 0.02 | 0.04 | 0.04 | 0.07 | 0.07 | 0.03 |
| LASSO | VAR | 0.073 |  |  |  |  |  |  |
| OKVAR-Boost | VAR | 0.034 | 0.02 | 0.05 | 0.05 | 0.03 | 0.02 | 0.02 |
| SWING-Lasso | VAR | 0.064 | 0.01 | 0.07 | 0.06 | 0.06 | 0.06 | 0.07 |
