# Supplementary material for: Causal network inference from gene transcriptional time-series response to glucocorticoids
Source: PLoS Comput Biol. 2021 Jan 29;17(1):e1008223. doi: 10.1371/journal.pcbi.1008223 (PMC7875426; doi:10.1371/journal.pcbi.1008223)
Supplement: S3 Table — AUPR, AUROC, and Time indicate average AUPR, AUROC, and time over the five networks, respectively. BETS and Enet are in bold to indicate that they are our own developed methods, based on vector autoregression. SWING-RF [51] and Jump3 [36] are decision tree methods. CSId is a Gaussian process method [44]. CLR [27], MRNET [90], and ARACNE [29] are mutual information methods. SWING-Lasso is a vector autoregression method [51]. Related to Fig 2. (DOCX) [file pcbi.1008223.s005.docx]

**S3 Table. Results of In-House Algorithms on DREAM4 100-gene Network Inference.** AUPR, AUROC, and Time indicate average AUPR, AUROC, and time over the 5 networks, respectively. BETS and Enet are bolded to indicate that they are our own developed methods, based on vector autoregression. SWING-RF [47] and Jump3 [32] are decision tree methods. CSId is a Gaussian process method [40]. CLR [23], MRNET [86], and ARACNE [25] are mutual information methods. SWING-Lasso is a vector autoregression method [47]. Related to Figure 2.

| **Method** | **AUPR**  **In-House** | **AUPR**  **Literature** | **AUROC**  **In-House** | **AUPR**  **Literature** | **Time (hours)**  **In-House** | **Literature Reference** |
| --- | --- | --- | --- | --- | --- | --- |
| SWING-RF | 0.212 |  | 0.772 |  | 0.11 | [47] |
| CSId | 0.208 | 0.234 | 0.728 | 0.712 | 9.8 | [45] |
| Jump3 | 0.182 | 0.187 | 0.72 |  | 45 | [32] |
| **BETS** | **0.128** |  | **0.688** |  | **4.8** |  |
| **Enet** | **0.098** |  | **0.662** |  | **1.2** |  |
| CLR | 0.072 | 0.123 | 0.678 | 0.699 | 0.0000089 | [36] |
| MRNET | 0.068 | 0.13 | 0.672 | 0.701 | 0.000011 | [36] |
| SWING-Lasso | 0.064 |  | 0.596 |  | 0.21 | [47] |
| ARACNE | 0.046 | 0.106 | 0.558 | 0.589 | 0.00001 | [36] |
