# Supplementary material for: Causal network inference from gene transcriptional time-series response to glucocorticoids
Source: PLoS Comput Biol. 2021 Jan 29;17(1):e1008223. doi: 10.1371/journal.pcbi.1008223 (PMC7875426; doi:10.1371/journal.pcbi.1008223)
Supplement: S4 Table — For each AUROC or AUPR column, the average is the listed value and the standard deviation is listed in parentheses. “Coefficient” denotes the result when ranking edges by their fitted coefficient, as in the original method. “Bootstrap” denotes the results when ranking edges by the frequency by which they appear in the bootstrap networks. (DOCX) [file pcbi.1008223.s006.docx]

**S4 Table. Improvement on DREAM4 100-gene Network Inference from Bootstrap.** For each AUROC or AUPR column, the average is the listed value and the standard deviation is listed in parentheses. "Coefficient" denotes the result when ranking edges by their fitted coefficient, as in the original method. "Bootstrap" denotes the results when ranking edges by the frequency by which they appear in the bootstrap networks.

| **Normalization** | **Lag** | **Penalty** | **Coefficient AUROC** | **Bootstrap AUROC** | **Coefficient AUPR** | **Bootstrap AUPR** |
| --- | --- | --- | --- | --- | --- | --- |
| 0mean | 1 | Elastic Net | 0.674 (0.05) | 0.686 (0.05) | 0.112 (0.03) | 0.14 (0.03) |
| 0mean | 2 | Elastic Net | 0.662 (0.05) | 0.688 (0.06) | 0.098 (0.02) | 0.128 (0.02) |
| 0mean | 2 | Lasso | 0.652 (0.05) | 0.692 (0.06) | 0.14 (0.04) | 0.162 (0.05) |
| 0mean | 2 | Ridge | 0.642 (0.04) | 0.66 (0.05) | 0.08 (0.03) | 0.096 (0.03) |
