# Supplementary material for: Causal network inference from gene transcriptional time-series response to glucocorticoids
Source: PLoS Comput Biol. 2021 Jan 29;17(1):e1008223. doi: 10.1371/journal.pcbi.1008223 (PMC7875426; doi:10.1371/journal.pcbi.1008223)
Supplement: S5 Table — DREAM results reported for running BETS on both 100 and 1000 bootstrap samples. All values in the columns are averages and the parenthetical values as standard deviations across the 5 DREAM4 Networks. The 1000 samples row is bolded because 1000 samples are the default settings. These use zero-mean normalization, lag 2, and the elastic net penalty. Related to Fig 2. (DOCX) [file pcbi.1008223.s007.docx]

**S5 Table. Dependency of BETS performance on Bootstrap Samples.** DREAM results reported for running BETS on both 100 and 1000 bootstrap samples. All values in the columns are averages and the parenthetical values as standard deviations across the 5 DREAM4 Networks. The 1000 samples row is bolded because 1000 samples are the default settings. These use zero-mean normalization, lag 2, and the elastic net penalty. Related to Figure 2.

| **Bootstrap Samples** | **AUROC** | **AUPR** | **Time (hr)** | **Memory (GB)** |
| --- | --- | --- | --- | --- |
| 100 | 0.68 (0.05) | 0.124 (0.02) | 1.6 | 1.6 |
| **1000** | **0.688 (0.06)** | **0.128 (0.02)** | **4.8** | **15.6** |
