# Supplementary material for: Causal network inference from gene transcriptional time-series response to glucocorticoids
Source: PLoS Comput Biol. 2021 Jan 29;17(1):e1008223. doi: 10.1371/journal.pcbi.1008223 (PMC7875426; doi:10.1371/journal.pcbi.1008223)
Supplement: S6 Table — A Fisher’s Exact Test was performed, where the rows of the contingency table were whether or not an edge was of the edge type, and the columns were whether or not the edge was part of the inferred network. Related to Fig 3. (DOCX) [file pcbi.1008223.s008.docx]

**S6 Table. Enrichment of edges between specific gene classes in inferred causal network.** A Fisher's Exact Test was performed, whether the rows of the contingency table were whether or not an edge was of the edge type, and the columns were whether or not the edge was part of the inferred network. Related to Figure 3.

| **Cause Type** | **Effect Type** | **Odds Ratio** | **Raw p-value** | **Adjusted p-value** | **Significant at FDR 0.05** |
| --- | --- | --- | --- | --- | --- |
| *TF* | *TF* | 1.32 | 4.43E-06 | 7.09E-06 | Yes |
| *TF* | *Imm* | 1.21 | 2.24E-02 | 3.25E-02 | Yes |
| *TF* | *Metab* | 1.14 | 7.54E-02 | 1.01E-01 | No |
| *TF* | *Other* | 1.21 | 4.82E-20 | 9.64E-20 | Yes |
| *Imm* | *TF* | 2.71 | 2.21E-45 | 1.18E-44 | Yes |
| *Imm* | *Imm* | 2.71 | 5.98E-23 | 1.60E-22 | Yes |
| *Imm* | *Metab* | 2.18 | 5.65E-14 | 1.01E-13 | Yes |
| *Imm* | *Other* | 2.66 | 0.00E+00 | 0.00E+00 | Yes |
| *Metab* | *TF* | 2.58 | 1.55E-43 | 6.20E-43 | Yes |
| *Metab* | *Imm* | 2.93 | 2.45E-30 | 7.85E-30 | Yes |
| *Metab* | *Metab* | 2.49 | 6.21E-22 | 1.42E-21 | Yes |
| *Metab* | *Other* | 2.94 | 0.00E+00 | 0.00E+00 | Yes |
| *Other* | *TF* | 0.87 | 1.00E+00 | 1.00E+00 | No |
| *Other* | *Imm* | 0.94 | 9.82E-01 | 1.00E+00 | No |
| *Other* | *Metab* | 0.86 | 1.00E+00 | 1.00E+00 | No |
| *Other* | *Other* | 0.69 | 1.00E+00 | 1.00E+00 | No |
