# Supplementary material for: Causal network inference from gene transcriptional time-series response to glucocorticoids
Source: PLoS Comput Biol. 2021 Jan 29;17(1):e1008223. doi: 10.1371/journal.pcbi.1008223 (PMC7875426; doi:10.1371/journal.pcbi.1008223)
Supplement: S7 Table — Shown Data Set indicates whether the gene temporal profiles in Fig 5 are taken from the original exposure data or unperturbed data. The edge type indicates the gene class of the causal and effect gene; for example, I → M indicates an edge from an Immune causal gene to a Metabolic effect gene. I = Immune; M = Metabolic; T = Transcription Factor; A = Any gene. Related to Fig 5. (DOCX) [file pcbi.1008223.s009.docx]

**S7 Table. Gene Pair information from Figure 5.** Shown Data Set indicates whether the gene temporal profiles in Figure 5 are taken from the *original exposure data* or *unperturbed data*. The edge type indicates the gene class of the causal and effect gene; for example, I → M indicates an edge from an Immune causal gene to a Metabolic effect gene. I = Immune; M = Metabolic; T = Transcription Factor; A = Any gene. Related to Figure 5.

| **Edge Type** | **Cause Gene** | **Effect Gene** | **Lag** | **VAR Coefficient** | **Average Coefficient** | **Selection Frequency** | **Shown Dataset** | **Correct Direction?** | **Citation** |
| --- | --- | --- | --- | --- | --- | --- | --- | --- | --- |
| I → I | TNFAIP3 | IRAK2 | 1 | 0.04 | 0.01 | 0.163 | unperturbed | N | [87] |
| I → M | SOCS1 | IRS2 | 1 | 0.1 | 0.01 | 0.1 | unperturbed | Y | [53, 55] |
| I → T | FOS | ATF3 | 1 | 0.16 | 0.1 | 0.709 | unperturbed | Y | [88] |
| I → A | FOS | HSPA1A | 2 | -0.06 | 0.02 | 0.2 | original | Y | [89] |
| M → I | IGFBP3 | CD44 | 1 | 0.03 | 0.01 | 0.212 | original | Y | [90] |
| M → M | SOCS3 | IRS2 | 2 | -0.04 | 0.01 | 0.099 | original | Y | [54] |
| M → T | SOCS3 | HIVEP1 | 1 | 0.07 | 0.02 | 0.297 | unperturbed | N | [91] |
| M → A | ATF3 | MDM2 | 1 | 0.06 | 0.02 | 0.46 | original | N | [92] |
| T → I | E2F1 | CDH1 | 1 | -0.04 | -0.01 | 0.12 | unperturbed | N | [93] |
| T → M | NR4A1 | RXRA | 2 | -0.04 | 0.02 | 0.471 | unperturbed | Y | [56, 57, 94] |
| T → T | BHLHE40 | HIVEP1 | 1 | 0.05 | 0.01 | 0.152 | original | Y | [91] |
| T → A | NR4A1 | VHL | 2 | 0.02 | 0.01 | 0.237 | unperturbed | Y | [95] |
| A → I | FOS | EGFR | 1 | 0.01 | 0.004 | 0.109 | original | N | [96] |
| A → M | ZFP36 | YWHAH | 2 | 0.01 | 0.004 | 0.131 | unperturbed | Y | [97] |
| A → T | NR0B1 | ESRRA | 1 | -0.04 | -0.004 | 0.102 | unperturbed | Y | [98] |
| A → A | CCNE2 | CDK2 | 1 | 0.07 | 0.04 | 0.636 | original | Y | [99–102] |
