# Supplementary material for: Causal network inference from gene transcriptional time-series response to glucocorticoids
Source: PLoS Comput Biol. 2021 Jan 29;17(1):e1008223. doi: 10.1371/journal.pcbi.1008223 (PMC7875426; doi:10.1371/journal.pcbi.1008223)
Supplement: S1 Text — Additional overview and analyses. (DOCX) [file pcbi.1008223.s010.docx]

**S1 Text. Supplemental Information.** Additional overview and analyses.

**Network inference methods**

Several methods have been developed to estimate directed graphs of genes from transcription time-series data (Fig S1). Broadly, these methods estimate directed networks in which the directed edges between nodes---representing genes---indicate a cause-effect relationship between those genes, such that perturbing the expression levels of the causal gene would lead to changes in expression of the effect gene [20].

Let *G* be the set of all genes and *g* be a single gene. Let *¬ g* be *G* with *g* removed. Let there be *T* time points total, and let *t* be a single time point ranging from {1, 2, …, T}. Let $X_{t}^{g}$ be the expression of gene *g* at time *t*. Let $\varepsilon_{t}$ be the residual noise at time *t*. Let : denote sequencing through values, for example $X_{1:T}^{g}$ would denote all the values $X_{1}^{g}$ through $X_{T}^{g}$. Let $pa(X_{t}^{g})$ refer to the causal parents of gene *g* at time *t* in dynamic Bayesian Networks. For example, $pa(X_{t}^{g})$ may include $X_{t-l}^{g'}$. Let *g'* be the gene we are testing to be causal for gene *g*. Let *l* be the time lag of the causal interaction. We are testing the existence of the edge $g^{'}\to g$ at lag *l*.

**Mutual information**

Mutual information (MI) methods assess the MI between the expression of *g'* at the *l-*th previous time point and the expression of *g* at the current time point (Fig S1A) [21-25]:

$$I^{l}(g^{'},g)= \sum_{t=l+1}^{T} P\left( X_{t-l}^{g^{'}},X_{t}^{g} \right)\log\frac{P\left( X_{t-l}^{g^{'}},X_{t}^{g} \right)}{P\left( X_{t-l}^{g^{'}} \right)P\left( X_{t}^{g} \right)}.$$

A causal edge $g^{'}\to g$ is included if $I^{l}(g^{'},g)$ exceeds a threshold. MI methods have the advantage of being simple and fast. However, they do not give insight into the sign of two genes' relationship (i.e., activation or repression) because MI is an unsigned metric [24, 26].

**Granger causality**

Granger causality methods determine if including the expression of *g'* at the previous time point improves our ability to predict the expression of *g* at the current time point above using the expression of *g* at the previous time point [27]. A common way to implement a Granger causality approach uses a vector autoregression (VAR) model, which usually assumes a linear relationship between all genes' previous expression and *g*'s current expression. (Fig S1B) [87].

$$X_{t}^{g}= \sum_{l=1}^{L} \alpha_{l}^{g}X_{t-l}^{g}+\sum_{g'\in g} \sum_{l=1}^{L} \beta_{l}^{g^{'}, g}X_{t-l}^{g'}+ \varepsilon_{t}.$$

A causal edge $g^{'}\to g$ is included in the network if $\beta_{l}^{g^{'}, g}$ is significantly different from 0 for some *l*. While older VAR analyses did not fit the causal predictors simultaneously [87-89], newer analyses fit them simultaneously, using regularization techniques such as lasso [13, 90] or ridge regression [91] to handle the high dimensionality of genome-wide sequencing assays. Ensemble methods have also been used to improve the performance of lasso [47]. Random forests and nonlinear, kernel-valued functions have also been used to implement ideas in Granger causality [47, 92].

**Ordinary differential equations**

Ordinary differential equations (ODEs) fit the derivative of the expression of *g* as a function of all genes' expression at a single time point (Fig S1C) [11, 28, 29]:

$$\frac{dX_{t}^{g}}{dt}=f\left( X_{t}^{g},X_{t}^{{g^{'}}_{1}},\ldots,X_{t}^{{g^{'}}_{G-1}} \right)+\varepsilon_{t}.$$

Although complex dynamics are often nonlinear, ODE methods typically assume linearity, as small sample sizes make it challenging to infer the parameters of nonlinear functions. A causal edge $g^{'}\to g$ is included in the network if *g'* has a significant coefficient in the ODE.

These methods are often combined with additional methods such as spline interpolation and piecewise linear functions to improve performance [28, 29].

**Decision trees**

Decision trees (DT) are a type of nonparametric function based on partitioning the data [30, 31]. DT methods fall either under VAR or ODE methods. Either the DTs fit the expression of *g* at the current time as a function of all genes' expression at the previous time point (VAR), or they fit the derivative of the expression of *g* as a function of all genes' expression at a single time point (ODE) (Fig S1D) [32, 33].

$$X_{t}^{g}=f\left( X_{t-1:t-L}^{g},X_{t-1:t-L}^{{g^{'}}_{1}:{g^{'}}_{\left| G \right|-1}} \right)+\varepsilon_{t}, f\in DT.$$

$$\frac{dX_{t}^{g}}{dt}=f\left( X_{t}^{g},X_{t}^{{g^{'}}_{1}}\ldots X_{t}^{{g^{'}}_{\left| G \right|-1}} \right)+\varepsilon_{t}, f\in DT.$$

A causal edge $g^{'}\to g$ is included in the network when an importance score for *g'*--- typically, the reduction in variance of *g* from including *g'* as a predictor--- exceeds some threshold. One limitation of DT methods is that they only produce a ranking of edges, without specifying the sign of the relationship between the genes [32, 33].

**Dynamic Bayesian networks**

Dynamic Bayesian networks (DBNs) search the space of possible directed acyclic graphs between previous and current expression levels and identify the network structure with the highest posterior probability of each edge given the data (Fig S1E) [34-38]. DBNs typically assume a linear relationship between previous expression values and current expression values. A causal edge $g^{'}\to g$ is included in the network when its marginal posterior probability of existence exceeds some threshold. The joint probability of all the genes' expression across the time points 1 : *T* factorizes as:

$$P\left( X_{1:T}^{g_{1}:g_{\left| G \right|}} \right)= \prod_{g\in G} P(X_{1}^{g})\prod_{t=2}^{T} P(X_{t}^{g}|pa\left( X_{t}^{g} \right)).$$

Each gene's expression has a linear relationship with its parents:

$$X_{t}^{g}=f\left( pa\left( X_{t}^{g} \right) \right)+ \varepsilon_{t}, f\in LINEAR.$$

While DBNs have been shown to be effective on smaller data sets [93], they scale poorly due to the superexponential growth of possible causal graph structures [58, 91]. Even after limiting the number of possible parents per gene to two, this results in cubic scaling of the search space. One exception is ScanBMA, which uses a pruning method based on Occam's window to limit the search space and gain a speedup [36].

**Gaussian process**

The Gaussian process (GP) is a distribution over continuous, nonlinear functions. GPs are often used in the context of nonlinear DBNs, where GP regression is used to model a nonlinear relationship between previous expression levels and current expression levels (Fig S1F) [39, 40]. A causal edge $g^{'}\to g$ is included in the network based on its posterior probability of existence, i.e., the sum of the posterior probabilities of those networks that contain the edge. Each gene's expression has a nonlinear relationship with its parents:

$$X_{t}^{g}=f\left( pa\left( X_{t}^{g} \right) \right)+ \varepsilon_{t}, f\sim GP.$$

By allowing nonlinear relationships between genes, GPs have proven highly effective. However, like DBN, they perform a search over causal graphs, and therefore suffer from the same scalability issues [39, 40].

**Sensitivity analysis of inferred network**

To assess the robustness of our inferred network to the choice of lag, we ran BETS with lag *L* = 1 on the glucocorticoid data. The discovered network has 2098 edges and shares 1286 edges with the lag *L* = 2 network's 31,945 edges. The overlap is significant (odds ratio (OR) = 393.9, Fisher's exact test (FET) $p\leq2.2* {10}^{-16}$ ). We validated the discovered network on the GTeX eQTL study and find 76 trans-eQTLs in all samples corresponding to 24 network edges. 74 of the trans-eQTLs pairs are in lung samples, corresponding with 22 network edges (q-value FDR $\leq0.2$). The remaining 2 trans-eQTLs were in adipose samples, corresponding to the 2 remaining network edges. There were 5 shared trans-eQTLs (4 network edges) between the 76 trans-eQTLs (24 network edges) discovered by lag *L* = 1 and 873 trans-eQTLs (341 network edges) discovered by lag *L* = 2. Thus, changing from lag 2 to lag 1, BETS still infers similar edges and discovers similar trans-eQTLs in lung tissue.

**Validation of inferred network on overexpression data**

Our analyses regressed network edge signs as predictors against the VAR model edge coefficients from the overexpression data as response (Fig 6). We sought to assess the strength of these associations across the 10 data sets, compared against shuffled edges. We compared the effect sizes of all 10 regressions of positive edge one-hot encodings on the overexpression coefficients with the effect sizes estimated similarly after shuffling the edge labels; we did the same for negative edges. At FDR $\leq0.2$, there was a substantial enrichment of effect sizes of positive edges among the original network (Common Language Effect Size (CLES) = 0.93, two-sided Mann-Whitney U-test (MWU) adjusted $p\leq0.0026$); there was no enrichment of effect sizes for negative edges in the original network (CLES = 0.55, two-sided MWU adjusted $p\leq0.73$). Thus, the positive edges inferred by BETS validate on the overexpression data, but the negative edges do not, indicating repressive effects may have inconsistent signs.

**Validation of BETS' prediction of targets of overexpression genes**

We generated a set of pseudo-targets for each overexpressed transcription factor $tf\in\{CEBPB, CEBPD, FOSL2, FOXO1, KLF15, KLF6, KLF9, OCT4, TFCP2L1\}$. For every gene $g\in G$, we use *nsgp* [94] to detect whether *g* is differentially expressed in the TF overexpression data set compared to the original exposure data set. The degree of *g*'s differential expression is measured by

$$\Delta_{MLL}^{tf,g}={MLL}_{ind}^{tf,g}-{MLL}_{joint}^{tf,g}.$$

Here, ${MLL}_{ind}^{tf,g}$ is the marginal log-likelihood of fitting one nonstationary GP for $X_{t}^{g}$ in the TF overexpression data set, and fitting an independent nonstationary GP for $X_{t}^{g}$ in the original exposure data set. ${MLL}_{joint}^{tf,g}$ is the marginal log-likelihood of fitting the same nonstationary GP over $X_{t}^{g}$ jointly in the TF overexpression data set and original exposure data set.

*nsgp* does not provide a framework for significance thresholding. We developed a procedure for generating a null distribution $\Delta_{MLL}^{tf,g}$ based on the null hypothesis is that *g* is not differentially expressed in the TF overexpression data set compared to the original overexpression data set. We replace the $X_{t}^{g}$ in the TF overexpression data set with a null version $\breve{X}_{t}^{g}$. $\breve{X}_{t}^{g}$ is created from $X_{t}^{g}$by randomly substituting an original exposure measurement $X_{t}^{g}$for an overexpression measurement of$X_{t}^{g}$at every time point *t* in the overexpression data set. We then compute $\breve{\Delta}_{MLL}^{tf,g}$as if $\breve{X}_{t}^{g}$ was the measurement in the TF overexpression data. We do this ten times, generating $\breve{\Delta}_{MLL,n}^{tf,g}$ where n $\in$ {1, 2, …, 10}.

We control the FDR at $\leq$ 0.01 by finding the threshold $T^{tf}$ such that

$$\frac{\frac{1}{10}\sum_{n=1}^{10} \sum_{g\in\neg tf} \boldsymbol{1}\{\breve{\Delta}_{MLL,n}^{tf,g}>T^{tf}\}}{\sum_{g\in\neg tf} \boldsymbol{1}\{\Delta_{MLL}^{tf,g}>T^{tf}\}+\frac{1}{10}\sum_{n=1}^{10} \sum_{g\in\neg tf} \boldsymbol{1}\{\breve{\Delta}_{MLL,n}^{tf,g}>T^{tf}\}}\leq0.01.$$

For every gene $g\in tf$, a directed edge $tf\to g$ exists (i.e., *g* is differentially expressed between the TF overexpression data set and original exposure data set) if $\Delta_{MLL}^{tf,g} > T^{tf}$.

Using this thresholding procedure, we find 443 targets of *CEBPB*, 318 targets of *CEBPD*, 223 targets of *FOSL2*, 342 targets of *FOXO1*, 19 targets of *FOXO3*, 767 targets of *KLF15*, 19 targets of *KLF6*, 212 targets of *KLF9*, 522 targets of *OCT4*, and 20 targets of *TFCP2L1*.

BETS is somewhat accurate at predicting the targets of *KLF15* (AUPR = 0.28), OCT4 (AUPR = 0.19), *CEBPB* (AUPR = 0.17), *FOXO1* (AUPR = 0.13), and *CEBPD* (AUPR = 0.11). It does not accurately predict the targets of *FOSL2*, *FOXO3*, *KLF6*, *KLF9*, and *TFCP2L1* (AUPR < 0.1).

Thus, BETS weakly predicts targets of 5 of the 10 over-expression TFs called by *nsgp*.

We generated a null by ranking every gene $g\in tf$ with a random score drawn from the uniform distribution on [0,1). We did this 10 different random seeds. The null has very similar predictive performance as BETS. The null somewhat accurately predicts the targets of *KLF15* (AUPR = 0.27-0.3), *OCT4* (AUPR = 0.18-0.21), *CEBPB* (AUPR = 0.15-0.17), *FOXO1* (AUPR 0.11-0.13), and *CEBPD* (AUPR = 0.11-0.12). It does not accurately predict the targets of *FOSL2*, *FOXO3*, *KLF6*, *KLF9*, and *TFCP2L1* (AUPR < 0.1).

Thus, random guessing also weakly predicts targets of 5 of the 10 over-expression TFs called by *nsgp*. BETS does not substantially improve over random guessing at FDR 0.1.
