# Supplementary material for: Causal network inference from gene transcriptional time-series response to glucocorticoids
Source: PLoS Comput Biol. 2021 Jan 29;17(1):e1008223. doi: 10.1371/journal.pcbi.1008223 (PMC7875426; doi:10.1371/journal.pcbi.1008223)
Supplement: Attachment — Submitted filename: BETS_r2r.pdf [file pcbi.1008223.s011.pdf]

Response to Reviewer Comments

We thanks the Reviewers for their helpful comments. We have made a large number of changes to the manuscript in response to these comments. Reviewer comments in italics; our point-by-point response in plain text below.

Reviewer 1 Comments and Responses

Reviewer #1: This manuscript presents the Bootstrap Elastic net regression from Time Series (BETS) algorithm, an network inference approach for time series gene expression data. The core question in time series-based network inference is how to use past information from regulators to detect their influence on the later expression levels of target genes. BETS adopts a Granger causality approach to address this question. Although Granger causality has been widely applied to time series gene expression data, there are multiple characteristics that differentiate BETS from prior work. The most notable is that BETS provides a false discovery rate (FDR) framework to rank and select network edges. Because it operates with temporal data, permuting the time points generates a convenient dataset in which the true regulator-target dependencies should be destroyed. Running BETS on the permuted data then creates a null distribution of regression coefficients or edge frequencies that can be used for FDR calculations. This FDR evaluation is combined with traditional bootstrapping and stability selection to make BETS robust to small sample sizes and false positive associations that are problematic in short time series. In addition, BETS incorporates an elastic net regression penalty and automated hyperparameter tuning, which are not implemented in many prior Granger causality methods.

In general, the presentation (figures, writing, review of related work, etc.), methodological choices, and analyses are superb. The synthesis of different types of time series network inference techniques in Figure S1 is an excellent high-level overview of the field. The evaluation on the simulated data DREAM4 challenge is valuable, demonstrating that BETS performs reasonably well on this popular benchmarking dataset and putting BETS in the context of many other types of network inference tools. There is a sizeable AUPR performance gap between BETS and the top method. However, this is not a concern because there are other advantages of BETS besides its AUPR on a simulated data set (e.g. parallelism, speed, and statistical rigor). Furthermore, other work in the gene network inference field has shown that AUPR on simulated data does not reflect the challenges of network inference in real mammalian systems, so maximizing AUPR on the DREAM4 data should not be the main objective of new methods.

Therefore, the glucocorticoid case study is more relevant and interesting. Because there are not complete gold standard networks available for evaluating condition-specific human regulatory networks, the BETS predictions are assessed with two independent datasets. Overexpression of 10 transcription factors demonstrates that there is general agreement between the gene expression changes induced by overexpression and the edge signs predicted by BETS. The

BETS predictions are not perfect, as four transcription factors have positive effects enriched among negative predicted edges. In addition, trans-eQTL analysis of GTEx lung gene expression data reveals many new trans-eQTLs that are nominated by the BETS network edges. These demonstrate how BETS predictions can be used to gain biological insights.

One weakness with respect to the method's originality and relevance to the network inference field is that there is not a direct analysis demonstrating that the novel methodological aspects of BETS have a practical impact. The DREAM4 results are 10 years old and do not reflect the state of the art in Granger causality analysis. Only BETS was run on the glucocorticoid express data. Conceptually, the null distributions and FDR-based approach should improve network quality, but this claim is not directly assessed.

Major comments:

Reviewer 1 Comment 1: *Related to the comment above, the manuscript would be improved by more specifically demonstrating to readers why they should use BETS over other modern Granger causality approaches. For instance, if the FDR framework is the main appeal, can it directly demonstrate the advantages of having a principled way to select the size of a network? If it is the scalability and parallelization, can the high-throughput software pipeline be made more robust and user friendly (see below)? There is a comparison with elastic net regression without stability selection, but ensembling or stability selection is now common place in network inference. The most closely related Granger causality work that shares some features with BETS is not included in the DREAM4 benchmarking. Examples of closely related methods include the last two methods in Section 2.1.6 of the supplement (the references are broken so specific manuscripts are unknown) or SWING (Finkle 2018 doi:10.1073/pnas.1710936115), which was not referenced.*

Author Response:

- As you suggested, we have performed a comparison against two other ensemble-based Granger causality methods: SWING-RF *(Finkle et al. 2018)* and SWING-Lasso *(Finkle et al. 2018)* on the DREAM data. SWING-RF is the top-performer of all 24 methods on the DREAM data set. However, SWING-RF is based on decision trees instead of vector autoregression, and therefore lacks interpretability of edges as being activating or inhibitory. It also does not have a thresholding approach for declaring a significant network. SWING-Lasso, on the other hand, is based on vector autoregression. However, it performs worse than BETS, achieving an average AUPR of 0.064 compared to BETS' 0.128 and an average AUROC of 0.596 compared to BETS' 0.688.

  We have now referenced SWING and these results in the main manuscript.

- In our initial submission, we had compared BETS against two other modern Granger causality frameworks implemented using vector autoregression: OKVAR-Boost (Lim 2013)

and Granger Causal Connectivity toolbox (Seth 2010). BETS outperforms all of them on DREAM, as in Figure 2A and in the 3rd paragraph under “Leading Performance on DREAM Network Inference Challenge.”

- An outdated supplement was uploaded in addition to the supplement already at the end of the main manuscript. We apologize for the confusion. We have fixed broken references in the new supplement.

Reviewer 1 Comment 2: *The analyses all fix the lag at $L = 2$. It is unknown whether that lag would still be appropriate for time-series data with more time points. The lag can be set by the user, but the relationships between the lag hyperparameter, the length of the time series, and the effectiveness of the FDR procedures have not been assessed.*

Author Response:

- We ran BETS with lag $L = 1$ on DREAM (Table S4) and find similar performance to lag $L = 2$. It achieves AUROC of 0.686 (compared to lag $L = 2$'s result of 0.688) and AUPR of 0.14 (compared to lag $L = 2$'s result of 0.12). We have added a paragraph under “Leading Performance on DREAM Network Inference Challenge” to highlight these results.

- Based on these comments, we have run BETS with lag $L = 1$ on the glucocorticoid data (Supplemental Information Section: “Sensitivity Analysis”). The discovered network has 2098 edges and shares 1286 edges with the lag $L = 2$ network. The overlap is significant (odds Ratio (OR) = 393.9, Fisher's exact test (FET) $p \leq 2.2 \times 10^{-16}$). We validated the discovered network on GTeX study data and found 76 trans-eQTLs corresponding to 24 network edges. 74 of the trans-eQTLs were in lung samples, corresponding to 22 network edges (q-value FDR ≤ 0.2). The remaining 2 trans-eQTLs were in adipose samples, corresponding to the 2 remaining network edges. 5 of the 74 discovered trans-eQTLs overlapped with the 341 trans-eQTLs discovered by lag $L = 2$, corresponding to 4 shared network edges. Thus, while using lag $L = 1$ reduced network size, the consistency and validity of the edges was still high.

- Assessing how time-series length and choice of lag jointly affect performance would require finding another biological data set of different time-series length and clear options for out-of-sample validation, as in our glucocorticoid data. This is beyond the scope of this work. We chose lag $L = 1$ and $L = 2$ due to the short nature of our time series.

Reviewer 1 Comment 3: *The permutation and bootstrapping concepts in Figure 1 are understandable at a high level. The methodological details are challenging to follow. It appears that a single temporal permutation is made in the outer loop and another is made in the inner loop. Is a single permutation sufficient to obtain a robust null distribution? In addition, if the*

bootstrapping is done on the matrix form in Equation 10, which has already unrolled or expanded the L previous time points, how is the inner loop permutation performed? The details of the permutation and FDR procedures are difficult to verify.

Author Response:

- We think a single permutation provides a robust null distribution because each gene is independently shuffled across time. Furthermore, if there are p genes, a single permutation would generate about $p(p-1)$ null coefficients. In the glucocorticoid data, this would be $2768 \times 2767 = 765906$ null coefficients.

- The updated Methods now explain in detail the inner loop permutation and FDR for the bootstrapped versions

  Refer to the original data set as X_t^g, the inner-loop permuted data set as $\tilde{X}_t^g$ (defined under "Permuted Coefficients" in Methods), and the outer-loop permuted data set as $\hat{X}_t^g$ (defined under "Stability FDR" in Methods). Let $\mathbf{X}_t^g$ and $\mathbb{X}_{t-\ell}^G$ be the "unrolled" output and input of the regression model in equation 10, listed again below.

$$\mathbf{X}_t^g = \mathbb{X}_{t-\ell}^G \bar{\beta}^g + \boldsymbol{\epsilon}_t.$$

  $\mathbf{X}_t^g$ is a $N \times 1$ vector and $\mathbb{X}_{t-\ell}^G$ is a $N \times L|G|$ matrix, where $N = R(T-L)$.

  Let $\tilde{\mathbf{X}}_t^g$ and $\tilde{\mathbb{X}}_{t-\ell}^G$ be the unrolled output and input, constructed from $\tilde{X}_t^g$. Let $\hat{\mathbf{X}}_t^g$ and $\hat{\mathbb{X}}_{t-\ell}^G$ be the unrolled output and input, constructed from $\hat{X}_t^g$.

  Let the jth bootstrap sample from the original data be $\mathbf{X}_t^{g,j}$ and $\mathbb{X}_{t-\ell}^{G,j}$. Let the jth bootstrap sample from the inner-loop permuted data be $\tilde{\mathbf{X}}_t^{g,j}$ and $\tilde{\mathbb{X}}_{t-\ell}^{G,j}$. Let the jth bootstrap sample from the outer-loop permuted data be $\hat{\mathbf{X}}_t^{g,j}$ and $\hat{\mathbb{X}}_{t-\ell}^{G,j}$.

  To generate bootstrap sample j, a set of N row indices, I^j, are sampled with replacement from $[1, 2, \ldots, N]$. $\mathbf{X}_t^{g,j}$ and $\mathbb{X}_{t-\ell}^{G,j}$, are created by choosing the rows I^j of $\mathbf{X}_t^g$ and $\mathbb{X}_{t-\ell}^G$. Similarly, $\tilde{\mathbf{X}}_t^{g,j}$ and $\tilde{\mathbb{X}}_{t-\ell}^{G,j}$, are created by choosing the rows I^j of $\tilde{\mathbf{X}}_t^g$ and $\tilde{\mathbb{X}}_{t-\ell}^G$. $\hat{\mathbf{X}}_t^{g,j}$ and $\hat{\mathbb{X}}_{t-\ell}^{G,j}$, are created by choosing the rows I^j of $\hat{\mathbf{X}}_t^g$ and $\hat{\mathbb{X}}_{t-\ell}^G$.

  In summary, the same row indices are used for generating the bootstrap samples from the original, inner loop permuted, and outer loop permuted data sets. We hope this clarifies the procedure. We have modified the text to reflect these clarifications.

Reviewer 1 Comment 4: *Using the BETS network's edge signs to predict the vector*

*autoregressive model's edge coefficients is a creative way to use the overexpression data to validate the network that accounts for the edge signs. However, there are some unaddressed caveats with this approach. The overexpression data have fewer time points, so a simpler regression model is used (Equation 17). Any errors in that simple model's fit will confound the BETS network assessment. This approach may also ignore false negative edges in the BETS network. An alternative approach would be to focus on the edge directions in the BETS network by estimating the differentially expressed genes in each overexpression experiment using a temporally-aware statistical test. nsgp (*Heinonen et al. 2015*; doi:10.1093/bioinformatics/btu699) or a similar method may be able to accommodate the different number of time points in the original and overexpression data. Then, these genes can be treated as the targets of that regulator in a pseudo-gold standard network. The predicted edges for that regulator in the BETS network could be evaluated with a precision-recall curve, even if they have very few predicted target genes.*

Author Response:

- We agree that any error in the simple model's fit may confound the BETS network assessment and acknowledge that caveat in the Results. However, the approach does not ignore false negative edges in the BETS network. Any false negative edges in the BETS network are classified as "no edge" in the regression. We regress a one-hot encoding of the network's edge sign (i.e., positive versus no edge or negative sign; negative versus positive or no edge) as the predictor against the VAR model edge coefficients estimated from each of the overexpression time series as the response.

- Based on your recommendation, we have performed the temporally-aware statistical test, nsgp

  We generated a set of pseudo-targets for each overexpressed transcription factor tf. For every gene $g \in G$, we use *nsgp*

$$\Delta_{MLL}^{tf,g} = MLL_{ind}^{tf,g} - MLL_{joint}^{tf,g}, \tag{1}$$

  where $MLL_{ind}^{tf,g}$ is the marginal log likelihood of fitting one nonstationary GP for X_t^g in the TF overexpression data, and fitting an independent nonstationary GP for X_t^g in the original exposure data. $MLL_{joint}^{tf,g}$ is the marginal log likelihood of fitting the same nonstationary GP over X_t^g jointly in the TF overexpression data and original exposure data.

  nsgp does not provide a framework for significance quantification of differentially expressed genes. We thus developed a procedure for generating a null distribution $\breve{\Delta}_{MLL}^{tf,g}$. The null hypothesis is that g is not differentially expressed in the TF overexpression data set. We generated a null $\breve{X}_t^g$ for the TF overexpression data by randomly substituting an original exposure measurement of X_t^g for an overexpression measurement of X_t^g for every time point t. We then compute $\breve{\Delta}_{MLL}^{tf,g}$ as before. We do this 10 times, generating $\breve{\Delta}_{MLL,n}^{tf,g}$ where $n \in \{1, 2, \ldots, 10\}$.

We control the FDR at ≤ 0.01 by finding the threshold T^{tf} such that

$$\frac{\frac{1}{10}\sum_{n=1}^{10}\sum_{g\in\neg tf}1\{\breve{\Delta}_{MLL,n}^{tf,g}>T^{tf}\}}{\sum_{g\in\neg tf}1\{\Delta_{MLL}^{tf,g}>T^{tf}\}+\frac{1}{10}\sum_{n=1}^{10}\sum_{g\in\neg tf}1\{\breve{\Delta}_{MLL,n}^{tf,g}>T^{tf}\}}\leq 0.01.$$

For every gene $g \in \neg tf$, a directed edges $tf \to g$ exists (i.e., g is differentially expressed between the TF overexpression data and original exposure data set) if $\Delta_{MLL}^{tf,g} > T^{tf}$.

Using this thresholding procedure, we find 443 targets of *CEBPB*, 318 targets of *CEBPD*, 223 targets of *FOSL2*, 342 targets of *FOXO1*, 19 targets of *FOXO3*, 767 targets of *KLF15*, 19 targets of *KLF6*, 212 targets of *KLF9*, 522 targets of *OCT4*, and 20 targets of *TFCP2L1*.

BETS is somewhat accurate at predicting the targets of *KLF15* (AUPR $= 0.28$), *OCT4* (AUPR $= 0.19$), *CEBPB* (AUPR $= 0.17$), *FOXO1* (AUPR $= 0.13$), and *CEBPD* (AUPR $= 0.11$). It does not accurately predict the targets of *FOSL2*, *FOXO3*, *KLF6*, *KLF9*, and *TFCP2L1* (AUPR < 0.1).

Thus, BETS weakly predicts targets of 5 of the 10 overexpression TFs called by *nsgp*.

We generated a null by ranking every gene $g \in \neg tf$ a random score drawn from the uniform distribution on $[0, 1)$. We did this 10 different random seeds. The null has very similar predictive performance as BETS. The null somewhat accurately predicts the targets of *KLF15* (AUPR $= 0.27 - 0.3$), *OCT4* (AUPR $= 0.18 - 0.21$), *CEBPB* (AUPR $= 0.15 - 0.17$), *FOXO1* (AUPR $0.11 - 0.13$), and *CEBPD* (AUPR $= 0.11 - 0.12$). It does not accurately predict the targets of *FOSL2*, *FOXO3*, *KLF6*, *KLF9*, and *TFCP2L1* (AUPR < 0.1).

Thus, random guessing also weakly predicts targets of 5 of the 10 over-expression TFs called by *nsgp*. BETS does not significantly improve over random guessing at FDR 0.1.

A caveat of this validation is that it is limited only to edges with one of the 10 overexpression TFs as a cause. We have included this full set of results in our supplemental information.

Reviewer 1 Comment 5: *The author contributions imply that the RNA-seq data were generated as part of this study. If that is the case, the experimental protocols and methods are incomplete. In addition, the expression data should be made available.*

Author Response:

We apologize for the omission. The full accession numbers are now listed in the Methods under "Glucocorticoid gene expression data." The research group that generated the overexpression data recently responded to our request to upload those data to Gene Expression Omnibus

(GEO), and we just began the process, which we anticipate will be complete by the time we receive reviews for this first revision.

Minor comments: ***Reviewer 1 Comment 6:*** *The supplement and GitHub readme note that the time points must be approximately equally spaced. This is an important assumption that limits the applicability to irregular time series. It should be stated more clearly in the main text.*

Author Response:

We agree. Per your suggestion, we have now added this to the main text with "One should note that many of these model frameworks work best with similarly-spaced time points, as in the data sets in this paper." under "BETS: A vector autoregressive approach to causal inference of gene regulatory networks."

Reviewer 1 Comment 7: *The supplement describes the global and local null distributions and FDR approaches in excellent detail. It is difficult to link this discussion to the methods that were actually used in the main text. Stating where the global and local versions were used in the main text methods would help connect these discussions.*

Author Response:

An outdated supplement was uploaded in addition to the supplement already at the end of the main manuscript to clarify these connections. In the main manuscript Methods, we describe only the null and FDR that were used for BETS to avoid any confusion (equations 14 and 15).

Reviewer 1 Comment 8: *The main text does not explain the biological goals of the glucocorticoid study or why immune and metabolic genes are of interest. It would help guide readers if some of the well-written explanation from the supplement's Sections 1 and 2.3 was moved to the main text results.*

Author Response:

Thank you for the recommendation. We have added the following sentence to the introduction:

"Thus, understanding the immune, metabolic, and transcriptional effects of GC may enable the development of improved anti-inflammatory treatments without metabolic side effects. A recent study assayed A549 lung cells over 12 hours to characterize the downstream effects of GC

Reviewer 1 Comment 9: *The discussion notes that applying Granger causality to single-cell pseudotemporal data is a relevant related area. This is indeed an exciting future direction for BETS, but recent preprints have shown that pseudotimes may not have the same information for network inference as bona fide time series data (Qiu 2018 doi:10.1101/426981; Deshpande 2019 doi:10.1101/534834). That related work may guide readers who attempt to apply BETS to pseudotemporal data.*

Author Response:

Thank you for the recommendation. We have raised this potential challenge and cited those preprints in the discussion by stating "Recent preprints suggest some limitations to pseudotime information for network inference

---

Reviewer 1 Comment 10: *The methods describes BIOGRID PPI, which do not appear to have been used in the analyses described in the results.*

Author Response:

- BIOGRID PPI was used to find the set of experimentally validated interactions discussed in the paragraph starting "Our network identified known biological interactions ..." We have clarified this in that section.
- We have removed the phrase about the STRING interactions as that was not used in the results.

Reviewer 1 Comment 11: *The references in the supplement are broken*

Author Response:

An outdated Supplement was uploaded in addition to the supplement already at the end of the main manuscript. We have fixed any broken references in the correct Supplement at the end of the manuscript.

Reviewer 1 Comment 12: *The supplement refers to a method named VAR-GEN instead of BETS. Are these the same?*

Author Response:

An outdated Supplement was uploaded in addition to the supplement already at the end of the main manuscript. We greatly apologize for the confusion. VAR-GEN was renamed to Enet.

Reviewer 1 Comment 13: *Supplemental Figure 7 is difficult to understand. What are the indices and percentages?*

Author Response:

An outdated Supplement was uploaded in addition to the Supplement already at the end of the main manuscript. That incomplete figure was not intended to be included in our submission.

Reviewer 1 Comment 14: *It is commendable that the software pipelines are available on GitHub with an open source license. Some challenges in running the software are detailed below. In addition, the final version should be archived on Zenodo, Figshare, Software Heritage, or a comparable resource. The Google Drive materials could also be migrated to a permanent repository.* The support group `https://groups.google.com/forum/#!forum/bets-support` displayed an error "You do not have permission to access this content. (# 418)".

Author Response:

Thank you for these concrete suggestions. We have resolved the support group issue. Unfortunately, the Google Drive materials take up substantial memory (> 100 GB) for completeness and we are unaware of a Github or other permanent repository that could hold these materials for a reasonable cost and time.

Reviewer 1 Comment 15: *The supplement contains typos "we uses the" (page 9) and "multiple sclrosis patients" (page 29).*

Author Response:

An outdated supplement was uploaded, and the new Supplement corrects these mistakes.

Software comments: The software was tested on Windows 10 with Git for Windows (GNU bash, version `4.4.23(1)-release (x86_64-pc-msys))` *in the following Python 2 conda environment:*

```
$ conda create --name bets python=2.7 numpy=1.13 scipy=0.19 pandas=0.20 matplotlib scikit-lear
```

Python 2's end of life date is in 2020 and support will be dropped by several packages BETS requires (https://python3statement.org/). Porting the code to Python 3 is strongly recommended.

Overall, the software would be much easier to run if it adopted an establishing pipeline workflow. The five major steps require substantial user intervention even though everything could be automated after the options in `package_params_cpipeline.sh` are configured. This would also help with eventual cross-platform compatibility (currently macOS is supported) and formal testing to ensure the pipeline can run on a different system.

The BETS pipeline in `BETS_tutorial.md` did not work in the environment described above. After editing dozens of lines, it was possible to run the code through Step 3 (Fit the model on the original data), but then there were too many scripts to edit.

The most common incompatibilities were: - Assuming 'python2' instead of 'python' to run .py files in the shell scripts - The 'rB' argument when loading pickled files generated "ValueError: Invalid mode ('rB')". Removing the 'rB' worked. - The 'wB' argument when writing pickled files needed to be 'w' instead. - The paths to scripts combined different path separators / and - The 'module load' command is not needed to load Python in many systems (this does not terminate execution though) - Exporting environment variables as a way to configure BETS can be unreliable.

Author Response:

Thank you so much for your feedback and testing of our code. Per your suggestions, we have ported the entire package to Python 3, fixed the 'rB' and 'wB' errors (to 'rb' and 'wb' to allow pickle loading and dumping) and tested the tutorial. It should work for Python3 on Mac OS X.

Reviewer 2 Comments and Responses

Reviewer #2: In the manuscript, Engelhardt and colleagues describe a new method called Bootstrap Elastic net regression from Time Series (BETS) to infer causal gene networks from time-series gene expression data. BETS uses vector autoregression with elastic net regularization to infer causal relationships (directed edges) between genes. The authors

benchmark the performance of BETS by comparing their results against those from 21 other methods on the time-series data from the DREAM4 Network Inference Challenge. Assessed using previously used metrics of performance (AUPR, area under the precision recal curve; AUROC, area under the ROC), BETS' ranks 6th out of 22 in terms of the AUPR metric (0.13 vs the top-performing CSId @ 0.2) and ranks 3rd out of 17 in AUROC (0.7 vs the top-performing CSId @0.72). Compared to other top-performing methods, BETS is the fastest (2-10 times faster), making it an attractive alternative to the top-performing CSId and Jump3. The authors demonstrate the utility of BETS by applying it on a previously published time-series expression (RNA-Seq) data on A549 cells (human adenocarcinoma cell line) exposed to dexamethasone (synthetic glucocorticoid). BETS-inferred causal gene network is validated against orthogonal overexpression datasets.

The proposed method is technically sound, and manuscript is generally well written, concise and easy to read. As the authors correctly state, BETS' advantage over other methods is its speed, with performance comparable to the best-performing methods. BETS would be a nice addition to the arsenal of methods used to infer causal gene networks from time-series expression data. And, the authors have made their method available on GitHub.

Major Points

Reviewer 2 Comment 1: *With regards to the text related to the inferred Glucocorticoid response network (on page 8), where the authors describe the inferred network containing 2,768 nodes (genes): It is noted that all 2,768 genes are 'effect genes' (had an incoming directed edge), and 466/2,768 genes are 'causes' (causal?), defined as nodes with an outward directed edge. If all the genes have an out-degree (outgoing edge), I don't understand how the authors can define 466 genes that has both incoming and outgoing edge as causal. I would think that only those nodes with one or more out-going edges and no incoming edge are 'causal.' The authors need to explicitly clarify what their definition of 'causal gene' is because this raises questions about their 'causal gene network' definition.*

Author Response:

We agree. Based on your recommendation, we have clarified in our manuscript with "In Granger causality and dynamical systems more generally, a causal gene g' is allowed to have incoming directed edges because g' can be affected by the past value of another gene g'', and g' can have a causal effect on the later value of a gene g."

Reviewer 2 Comment 2: *The fact that all 2,768 genes have an out-going edge means that the resulting network/graph is not a directed acyclic graph (DAG; network free of cycles) and that it contains strongly connected component (SCCs), defined as a sub-networks where, for every pair*

of nodes u and v in the sub-network, there exists a directed path from u to v, and from v to u. Given this, I wonder if a method like Vertex Sort (PMID: 19690563) would be more appropriate to infer the network hierarchy and thus 'causal genes'.

Author Response:

Thank you for the recommendation. We agree that our inferred graph is not a DAG and contains strongly connected components, and we have modified the text to reflect this clearly. We have sought to clarify the issue in our main manuscript. However, there is not a readily available Vertex Sort software package (PMID: 19690563 only provides pseudocode), and we believe that the implementation and validation of Vertex Sort to causal networks would be out of the scope of this work.

Minor Points:

***Reviewer 2 Comment 1:**. I recommend the authors consider using "time-series" instead of "time series."*

Author Response:

We agree. We now use "time-series" when used as an adjective and "time series" when used as a noun.

---

2. The authors have been objective overall in describing/interpreting their results/findings. Line 184 on page 6 states that "BETS had a slightly lower AUPR compared with CSId (0.12 vs 0.20)." I take issue with 'slightly' since CSId's is 65% better than BETS w.r.t this metric. I would just remove 'slightly.'

Author Response:

We agree. We have removed 'slightly.'

Have all data underlying the figures and results presented in the manuscript been provided? Large-scale datasets should be made available via a public repository as described in the PLOS

Computational Biology data availability policy, and numerical data that underlies graphs or summary statistics should be provided in spreadsheet form as supporting information.

Reviewer #1: No: The manuscript states ”All files have been or are being submitted to the Gene Expression Omnibus under the same study name, including accession number GSE91208.” However, that accession number only lists DNase-seq data, not RNA-seq data. Reviewer access links to any private data used in this study should be made available.

Author Response:

We apologize for the omission. The full accession numbers are now listed in the Methods under “Glucocorticoid gene expression data.” For the overexpression data, we have just begun the process to upload those data to Gene Expression Omnibus, and will update the manuscript as soon as that is complete.
