# Supplementary material for: Causal network inference from gene transcriptional time-series response to glucocorticoids
Source: PLoS Comput Biol. 2021 Jan 29;17(1):e1008223. doi: 10.1371/journal.pcbi.1008223 (PMC7875426; doi:10.1371/journal.pcbi.1008223)
Supplement: Attachment — Submitted filename: bets_r2r_v2.pdf [file pcbi.1008223.s012.pdf]

Response to Reviewer Comments

We thank the Reviewers for their helpful comments, which we address here one-by-one. We have made a number of important changes to the manuscript and the software in response to these comments. Reviewer comments in italics; our point-by-point response in plain text below.

Reviewer 1 Comments and Responses

Reviewer #1: The authors have made substantial revisions that greatly enhance the manuscript and address almost all of my comments with the initial submission. These include clarifications in the Methods text, demonstration of robustness to the lag parameter values, results from new network inference algorithms, and another approach for evaluating the glucocorticoid predictions. Even though not all of the quantitative results favor the BETS algorithm, the additional results strengthen the manuscript. The authors have conducted fair and objective evaluations instead of skewing the results and language to only highlight advantages of BETS. For instance, they are honest in reporting that SWING-RF is now the top performer in the DREAM dataset and the nsgp-based glucocorticoid evaluation shows the BETS predictions are not significantly better than random guessing. (Those glucocorticoid predictions are still supported by two other analyses.) All network inference algorithms have some limitations, so the objective assessment will raise readers' confidence in the reported results and improves the manuscript overall.

The main reason I am enthusiastic about this manuscript is that the false discovery rate framework is a major and novel contribution to the network inference field. Figure 2 shows that BETS substantially improves upon other vector autoregressive network inference algorithms (including the new SWING-Lasso) in the DREAM evaluation. BETS remains very good at identifying trans-eQTLs. In addition, the analyses and results are rigorous, and the manuscript is very polished overall.

The only remaining major concern is the usability of the BETS software, as detailed below.

Major comments:

Reviewer 1 Comment 1: *I am still unable to run the BETS pipeline. This time I tried running BETS in Python 3 on a Linux server. I created a fresh conda environment with*

```
$ conda create --name bets python=3 numpy=1.13 scipy=0.19 pandas=0.20 matplotlib scikit-learn
```

Author Response:

We apologize for the issues. We have fixed the errors by running BETS in Python 3 on a Linux server with the same conda environment. We have verified that running all of the steps under BETS_tutorial.md is successful, and does not produce any more of the following errors.

There were fatal errors within the first few minutes of running the pipeline described in BETS_tutorial.md

- The line 'export NULL=g' comes after $NULL is used in package_params_cpipeline.sh so the directory names are incorrect

Author Response:

We have fixed the export NULL=g to be before the directory name is created and before all other usages of NULL.

- prep_jobs_bootstrap.sh still uses the 'python2' command to run the Python script so it does not work in a Python 3 environment. Because this command is run within a script, aliasing python2 to point to python3 does not work.

Author Response:

We have fixed to use python3. Thank you for catching that.

Because the software is an important part of the contribution, the authors should ensure the pipeline can run in a fresh Python environment. One way to guarantee this would be to run it in a continuous integration service like Travis CI or GitHub Actions.

Author Response:

We have verified that the pipeline can be run in the fresh Python3 conda environment that you shared on two separate linux servers.

Minor Points:

Reviewer 1 Comment 2: *To keep the discussion balanced, some of the new negative results could be included alongside the existing positive results. SWING-RF is very fast and accurate (Table S3), but it is not included in the runtime discussion on line 191. BETS is substantially faster than CSId and Jump3 but not SWING-RF. The Discussion paragraph starting at line 379*

only focuses on the positive validations for the glucocorticoid network but ignores the nsgp-based results

Author Response:

Thank you for catching both of our oversights.

We have discussed the negative nsgp-results in the Discussion paragraph, now starting at line 380. The editted sentences now read: "We validated our network first in ten overexpression data sets. Edges that were positive in the original network had an enrichment of positive VAR effect sizes in the overexpression data. However, edges in the original network did not predict differential expression of genes in the overexpression data, as called by*nsgp*."

We have included SWING-RF into the runtime and accuracy discussion, starting on line 191. We acknowledge that SWING-RF is both faster and more accurate than BETS. The section now reads: "we compared the speed of BETS and three other top-performing methods: SWING-RF, CSId and Jump3 (Table S3). SWING-RF was the fastest at 0.11 hours, while BETS took 4.8 hours, CSId took 9.8 hours and Jump3 took 45 hours. Thus, while BETS had a lower AUPR compared with CSId and Jump3, it was substantially faster than them. BETS had both a lower AUPR and longer runtime than SWING-RF.

Reviewer 1 Comment 3: *The editors should ensure the overexpression data is available on GEO before publication.*

Author Response:

The unperturbed data is available at GSE144662, while the overexpression data is currently available at GSE144660. We have clarified this in the manuscript, at line 585-585: "The unperturbed data set is available at GEO accession number GSE144662." and line 604-605: "The overexpression data sets are available at the Gene Expression Omnibus at GEO accession number GSE144660."

Instructions for access for peer reviewers:

1) Go to `https://www.ncbi.nlm.nih.gov/geo/query/acc.cgi?acc=GSE144662` for the unperturbed data and `https://www.ncbi.nlm.nih.gov/geo/query/acc.cgi?acc=GSE144660` for the over-expression data.

2) Enter token gfahosmetxcldif into the box.

Reviewer 1 Comment 4: *The source code and supplementary materials on Google Drive should be archived in a more permanent repository, even if they are ¿ 100 GB. The NIH figshare*

instance allows 100 GB of storage, and researchers can request more (https://nih.figshare.com/f/faq). Zenodo has 50 GB by default, but more is available upon request (https://about.zenodo.org/policies/). PLOS Computational Biology partners with Dryad (https://datadryad.org/stash/publishing_charges) and offers a 300 GB limit (https://datadryad.org/stash/faq)

Author Response:

We have uploaded the source code, data, analysis notebooks, figures and supplementary materials to Zenodo at `https://zenodo.org/record/3945082#.Xw31cpNKhn4` under "port-from-della.zip", with a reference for each figure and number produced in the paper under "FullProgeny.xlsx". We have also added it into the paper.

Reviewer 1 Comment 5: *Some inconsistencies and typos have been introduced over the different versions of the manuscript*

- References to 'STAR Methods' remain

- Some text describes 340 trans-eQTLs, other text states 341

- Line 442 states 'In BETS, L = 2.' but now the results include multiple values of L, so this could state L = 2 is the default

- Line 476 'that the the'

- Line 576 states 'reference numbers listed in Supplementary Table 3' but that table contains other data

- The 'DREAM benchmarking' section (line 601) omits the new SWING methods

- Line 638 still refers to STRING interactions

- Line 799 'gene $g \in \not{t}f$ a random score' is missing a word

Author Response:

We have fixed the errors:

- we removed "STAR" mentions

- we clarified that 341 gene network edges were validated in total, including 130 gene network edges in lung tissue (corresponding with 340 lung trans-eQTLs, which happens to look like the 341 gene network edges but that is a coincidence). We verified that there are no inconsistencies.

- we made the suggested edit in line 442 (now line 445)

- we fixed the typo in line 476

- we clarified that the corresponding reference numbers can be found under the column "Experiment_GEO_Series" based on rows that have "RNASeq" as the assay in Supplementary Table 3 in line 576 (now lines 580-581)

- we added the new SWING methods in at line 601 (now lines 609)

- we removed the STRING interactions mention in line 638

- we added "with" to fix the grammatical error in line 799 (now line 806)

Have all data underlying the figures and results presented in the manuscript been provided? Large-scale datasets should be made available via a public repository as described in the PLOS Computational Biology data availability policy, and numerical data that underlies graphs or summary statistics should be provided in spreadsheet form as supporting information.

Reviewer #1: No: Not yet. The accession number GSE91208 is listed but does not seem relevant. The GEO accession numbers for the 100 nM dexamethasone treatment time course have now been provided. The GEO uploads for the overexpression data are still in progress. The Google Drive data should still be archived as well.

Author Response:

The unperturbed data is available at GSE144662, while the overexpression data is currently available at GSE144660. We have clarified this in the manuscript, at line 585-585: "The unperturbed data set is available at GEO accession number GSE144662." and line 604-605: "The overexpression data sets are available at the Gene Expression Omnibus at GEO accession number GSE144660.

Instructions for access for peer reviewers:

1) Go to `https://www.ncbi.nlm.nih.gov/geo/query/acc.cgi?acc=GSE144662` for the unperturbed data and `https://www.ncbi.nlm.nih.gov/geo/query/acc.cgi?acc=GSE144660` for the over-expression data.

2) Enter token gfahosmetxcldif into the box.
